# Space-fractional quantum heat engine based on level degeneracy

**DOI:** 10.1038/s41598-021-97304-5

**Published:** 2021-09-09

**Authors:** Ekrem Aydiner

**Affiliations:** Department of Physics, Faculty of Science, İstanbul University, 34134 İstanbul, Turkey

**Keywords:** Biotechnology, Energy science and technology, Materials science, Nanoscience and technology, Physics

## Abstract

In order to examine the work and efficiency of the space-fractional quantum heat engine, we consider a model of the space-fractional quantum heat engine which has a Stirling-like cycle with a single particle under infinite potential well as an example. We numerically compute the work and efficiency for various fractional exponents. We show the work and the efficiency of the engine depending on the length of the potential well and fractional exponent of the engine. Furthermore, we show that fractional exponent plays a substantial role in the operating range of the quantum heat engine. Thus, we conclude that the fractional parameter can be used as a tuning parameter to obtain positive work and efficiency for the large size of the quantum heat engine. Additionally, the numerical results and model imply that the size of the engine can be enlarged in the nano-scale by using fractional deformations. As a result, in this study, we have not only shown that fractional deformations in space play an important role on the work and efficiency of the quantum heat engines but also introduced the concept of fractional quantum heat engines to the literature.

## Introduction

Firstly, the quantum version of the Szilárd engine was proposed by Kim et al. ^1^ as the counterpart of the classical one^2^. Later, in many theoretical studies, it has been shown that positive work and efficiency can be obtained by using quantum Szilard engine^3–8^. Furthermore, many experimental realizations have been implemented both of quantum engine and quantum Szilard engine^9–22^. It is well known that the Szilard engine converts information into useful work, and hence measurement is needed to extract work. However, recently, a similar version of the quantum Szilard engine has been studied by Thomas et al.^23^ for the infinite well potential based on level degeneracy. They proposed a quantum heat engine that has a Stirling cycle and it produces work that only depends on quantum features. They showed that, without any measurement, it could be converted into useful work by utilizing a quantum system whose energy levels degenerate and using two different reservoirs.

More recently, a quantum Szilard engine model for the fractional potential has been studied^24^ based on the previous model proposed by Thomas et al.^23^. However, the quantum heat engines and information theory have not been comprehensively studied by using fractional methods in the literature so far. It is known that fractional dynamics play an vital role in physics and other science areas^25–34^. Therefore, it is important to discuss and understand the role of fractional dynamics for the quantum heat engines. In the previous study^24^, it was obtained the work and efficiency of the model without using fractional calculus tools. However, in the present study, we discuss the fractional quantum Szilard engine as an example and we show that fractional exponent plays an important role in the work and efficiency. Therefore, in this study, we consider a quantum engine which has Stirling-like cycles and we discuss the work and efficiency based on the space-fractional Schrödinger equation for a single particle under the infinite well potential.

The remain of the paper is organized as follows: Firstly, we summarize the solution of the space-fractional Schrödinger equation for a single particle under the infinite potential well. Then, we discuss the theoretical framework of the thermodynamics cycles for the fractional quantum engine based on energy degeneracy. In subsequent section, we obtain the work and efficiency of the fractional quantum engine. We also discuss the effects of the space-fractional exponent on the work and efficiency. Finally, in the last section, we summarize the obtained results and give a brief discussion.

## Space-fractional Schrödinger equation

Before giving a brief discussion of the solution for the space-fractional Schrödinger equation, we would like to remind that the space or time-fractional processes can be caused by spatial deformation, entropic restrictions, or deformed potentials. It is well known that the differential equations which correspond to these processes can be represented with a non-integer derivative value^25–32^.

It is known that a discrete stochastic dynamics is categorized by the finite characteristic waiting time $T=\int _{0}^{\infty } t \psi (t) dt$ and the finite jump length variance $\Sigma ^{2} =\int _{-\infty }^{\infty } x^{2} \lambda (x) dx$ where $\psi (t)$ and $\lambda (x)$ respectively denote the waiting time and jump length probability distributions. For instance, for a Markovian process, $\psi (t)$ is of Poisson form and $\lambda (x)$ has Gaussian form. However, for the non-Markovian process, the waiting time *T* diverges, and the jump length variance $\Sigma ^{2}$ is still kept finite. In a such process, the long-tailed waiting time probability distribution takes an asymptotic form and which leads to the time-fractional equation in the continuum limit^30^. Conversely, in the other case, it leads to the space-fractional equation in the continuum limit. As a result, space and time exponents are represented by $\alpha$ and $\beta$, respectively. The space-fractional exponent is between $0<\alpha \le 2$, however, the time-fractional exponent is between $0<\beta \le 1$.

In a physical process, the value of these exponents which affect the solution of the differential equation can be controlled by changing the effects of the spatial deformation, entropic restrictions, or deformed potentials.

Now, to proceed discussion, we consider a one-dimensional space-fractional Schrödinger equation for a single particle in a box of length 2*a* under the infinite well potential. This problem was discussed by Laskin in several papers^32–34^. Here we briefly review the fractional Schrödinger equation and its results for infinite well potential. In the Refs. ^32–35^, the Hamiltonian of the system is given by

1 $$\begin{aligned} H_{\alpha } \psi _{n}(x) = E_{\alpha n} \psi _{n},(x) \quad n = 0,1,2,... \end{aligned}$$

where $\alpha$ denotes the fractional index $0 < \alpha \le 2$. For the infinite square well with a length of 2*a*, the space-fractional Schrödinger equation is given by

2 $$\begin{aligned} D_{\alpha } \left( - \hbar ^{2} \frac{d^{2}}{dx^{2}} \right) ^{\alpha /2} \psi _{n}(x) + V \psi _{n}(x) = E_{\alpha } \psi _{n}(x) \end{aligned}$$

where the coefficient $D_\alpha = \chi m c^{2} / (mc)^{\alpha }$ with $\chi$ a positive real number, and *c* is the speed of the light. When $\alpha =2$, taking $\chi =1/2$ and hence $D=1/(2m)$^35^. The first term in Eq. (2) corresponds to the fractional kinetic energy

3 $$\begin{aligned} T_{\alpha } = D_{\alpha } |p|^{\alpha } =\frac{1}{2} m c^{2} \left( \frac{ |p| }{m c} \right) ^{\alpha } = D_\alpha \left( - \hbar ^{2} \frac{d^{2}}{dx^{2}} \right) ^{\alpha /2} . \end{aligned}$$

On the other hand, the potential for infinite well is defined as

4 $$\begin{aligned} V = \left\{ \begin{array}{ll} 0 &{} \hbox { if}\ |x| < a \\ +\infty &{} \hbox { if}\ |x| > a \end{array} \right. \end{aligned}$$

the potential takes infinite value at the $x=\pm a$. Finally, the solutions of the fractional Schrödinger equation are given by

5 $$\begin{aligned} \psi _{n} (x) = \left\{ \begin{array}{ll} \frac{1}{\sqrt{2}}\sin \frac{n\pi }{2a}x + a&{} \hbox { if}\ |x| < a \\ 0 &{} \hbox { if}\ |x| > a \end{array} \right. \end{aligned}$$

and

6 $$\begin{aligned} E_{\alpha n} = D_{\alpha } \left( \frac{n \pi \hbar }{2 a} \right) ^{\alpha } \end{aligned}$$

where $\psi _{n} (x)$ denotes the wave function solution and $E_{\alpha n}$ is the energy eigenvalues of Eq. (2). Here we note that some authors claim that the solution does not satisfy the ground state solution of the space-fractional Schrödinger equation (2). However, it is shown that this problem can be solved by assuming that the solution is the limit of the finite square well problem^35^. Thus, the solution

7 $$\begin{aligned} \lim _{V_{0}\rightarrow \infty } \psi _{n}^{finite} (x) = \psi _{n} (x) \qquad \lim _{V_{0}\rightarrow \infty } E_{\alpha n}^{finite} = E_{\alpha n} \end{aligned}$$

satisfies the ground state requirement of the infinite square well^35^.

To carry out the work and efficiency of the fractional quantum Szilard like-engine we need the canonical partition function and heat exchanges for all stages. Therefore, in the next, we discuss these quantities for the infinite well potential given in Eq. (4) following the method given in Ref. ^23^.

## Stirling-like thermodynamics cycle

The presented model is the fractional box of length 2a containing a single molecule under infinite potential. To extract work, we consider the Stirling cycle which consists of four stages as well previous studies^23,24^. All stages and related physical quantities are obtained below.

**Figure 1:**
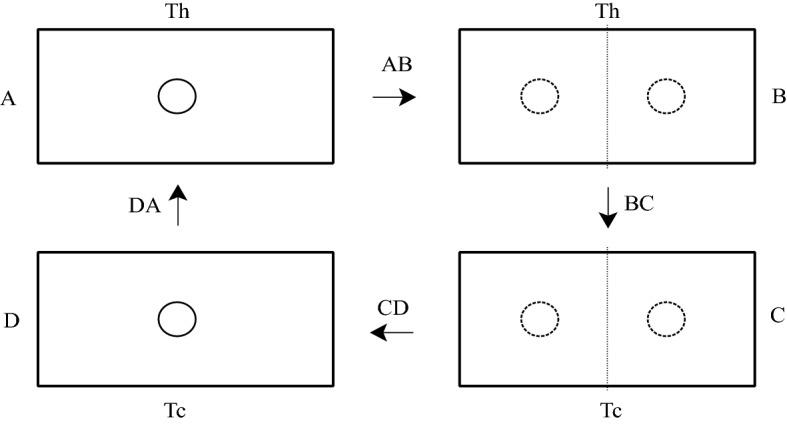
The sub-processes of the fractional quantum heat engine for the infinite well in a box of length 2a. In this schema boxes A and B interact a hot bath reservoir, boxes C and D interact a cold bath reservoir. The stages AB and CD represent the isothermal insertion and removal processes. The stages BC and DA denote the isochoric processes.

In Fig. 1, box A with a single particle in the infinite potential interacts with a heat bath with a higher temperature of $T_{h}$. However, in AB isothermal stage, a thin potential barrier is completely inserted in the box at the same temperature and the box is divided by the quasi-static insertion process into two half sides as in box B which behaves like the two double-well potentials.

In BC isochoric stage, the system is contacted to a heat bath with a lower temperature of $T_{c}$. However, in CD isothermal stage, the inserted thin barrier is again removed. After CD process, the particle is again under a single infinite well potential in box D with a cold bath temperature of $T_{c}$. In the final DA isochoric stage, the system is contacted to a heat bath with a higher temperature of $T_{h}$ and the cycle is completed.

The energy eigenvalue for the particle in box A and D is given by

8 $$\begin{aligned} E_{\alpha n}^{A,D} = D_{\alpha } \left( \frac{n \pi \hbar }{2 a} \right) ^{\alpha } . \end{aligned}$$

The canonical partition functions $Z_{A}(\alpha )$ and $Z_{D}(\alpha )$ for the particle in boxes A and D

9 $$\begin{aligned} Z_{A}(\alpha ) = \sum _{n=1}^{\infty } \exp \left[ -\frac{D_{\alpha } }{k_{B}T_{h}} \left( \frac{n \pi \hbar }{2 a} \right) ^{\alpha } \right] . \end{aligned}$$

and

10 $$\begin{aligned} Z_{D}(\alpha ) = \sum _{n=1}^{\infty } \exp \left[ -\frac{D_{\alpha } }{k_{B}T_{c}} \left( \frac{n \pi \hbar }{2 a} \right) ^{\alpha } \right] \ . \end{aligned}$$

On the other hand, the energy eigenvalue for boxes B and C is given as

11 $$\begin{aligned} E^{B}_{\alpha n} = D_{\alpha } \left( \frac{2 n \pi \hbar }{2 a} \right) ^{\alpha } . \end{aligned}$$

The correspondence partition functions $Z_{B}(\alpha )$ and $Z_{C}(\alpha )$ are given as

12 $$\begin{aligned} Z_{B}(\alpha ) = \sum _{n=1}^{\infty } 2 \exp \left[ - \frac{D_{\alpha }}{k_{B}T_{h}} \left( \frac{2 n \pi \hbar }{2 a} \right) ^{\alpha } \right] . \end{aligned}$$

and

13 $$\begin{aligned} Z_{C}(\alpha ) = \sum _{n=1}^{\infty } 2 \exp \left[ - \frac{D_{\alpha }}{k_{B}T_{c}} \left( \frac{2 n \pi \hbar }{2 a} \right) ^{\alpha } \right] \end{aligned}$$

where the pre-factor 2 in Eqs. (12) and (13) is written since the boxes are divided into two by the barrier which leads to energy levels two-fold degenerate.

On the other hand, the heat exchanges $Q_{AB}$, $Q_{BC}$, $Q_{CD}$ and $Q_{DA}$ for all stages of the thermodynamical cycle can be obtained by using the partition functions. For this isothermal process AB, the heat exchanged $Q_{AB}$ can be obtained as

14 $$\begin{aligned} Q_{AB} = U_{B} - U_{A} + k_{B} T_{h} \ln \frac{Z_{B}(\alpha )}{Z_{A}(\alpha )} \end{aligned}$$

where $U_{A}$ and $U_{B}$ are the internal energies of the box A and B, respectively, which can be obtained from

15 $$\begin{aligned} U_{A,B} = - \frac{\partial Z_{A,B}(\alpha )}{\partial \beta _{h}} \end{aligned}$$

where $\beta _{h}=1/k_{B}T_{h}$. Another isothermally stage CD leads to heat exchange $Q_{CD}$ which is written as

16 $$\begin{aligned} Q_{CD} = U_{D} - U_{C} + k_{B} T_{c} \ln \frac{Z_{D}(\alpha )}{Z_{C}(\alpha )} \end{aligned}$$

where $U_{C}$ and $U_{D}$ are internal energies for box C and D, respectively which can be found from

17 $$\begin{aligned} U_{C,D} = - \frac{\partial Z_{C,D}(\alpha )}{\partial \beta _{c}} \end{aligned}$$

where $\beta _{c}=1/k_{B}T_{c}$ is the inverse temperature. On the other hand, the heat exchanges for two isochoric processes BC and DA are given below. The amount of exchanged heats $Q_{BC}$ and $Q_{DA}$ during isochoric process BC and DA are given by

18 $$\begin{aligned} Q_{BC} = U_{C} - U_{B} \end{aligned}$$

and

19 $$\begin{aligned} Q_{DA} = U_{A} - U_{D} \ . \end{aligned}$$

By using the partition functions and heat exchanges we can compute work *W* and efficiency $\eta$.

## Work and efficiency of the fractional heat engine

The work for the Striling like cycle can be obtained from

20 $$\begin{aligned} W(\alpha ) = k_{B} T_{h} \ln \frac{Z_{B}(\alpha )}{Z_{A}(\alpha )} - k_{B} T_{c} \ln \frac{Z_{C}(\alpha )}{Z_{D}(\alpha )} \end{aligned}$$

and the efficiency is given by

21 $$\begin{aligned} \eta (\alpha ) = 1 + \frac{ Q_{BC} + Q_{CD} }{ Q_{DA} + Q_{AB} } \end{aligned}$$

which is defined in terms of the heat exchanges. In the calculation, for simplicity, we neglect some extra energy costs for instance needed energy for insertion and removal of the barrier and needed the energy for the coupling or decoupling of the system to the heat baths.

**Figure 2:**
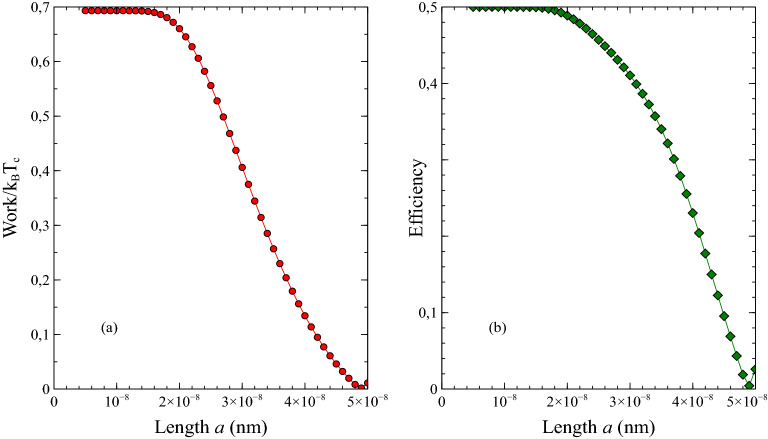
(**a**) Work, (**b**) Efficiency versus length *a* (nm) for the non-fractional quantum heat engine. Here, we have set the constants as $m=9.11\times 10^{-31}$ kg, $k_{B}=1.380649\times 10^{-23}$ J/K, $h=6.62607015\times 10^{-34}$ Js $T_{h}=2$ K and $T_{c}=1$ K.

As a result, by using Eq. (20) and Eq. (21) we numerically obtained the work *W* and the efficiency $\eta$ of a Stirling-like cycle presented in Fig. 1 for the $\alpha =2.0$. We note that for the $\alpha =2$, Eq. (6) reduce to

22 $$\begin{aligned} E_{n} = \frac{n^{2} \pi ^{2} \hbar ^{2}}{8m a^{2}} \end{aligned}$$

which is the energy eigenvalue of a particle in the infinite quantum well. The obtained numerical results for the $\alpha =2.0$ are given in Fig. 2. Both thermodynamic quantities in Fig. 2 are plotted as a function of the parameter *a* which denotes the width of each well of a double well potential. Here, we have set the constants as $m=9.11\times 10^{-31}$ kg, $k_{B}=1.380649\times 10^{-23}$ J/K, $h=6.62607015\times 10^{-34}$ Js, $T_{h}=2$ K and $T_{c}=1$ K.

**Figure 3:**
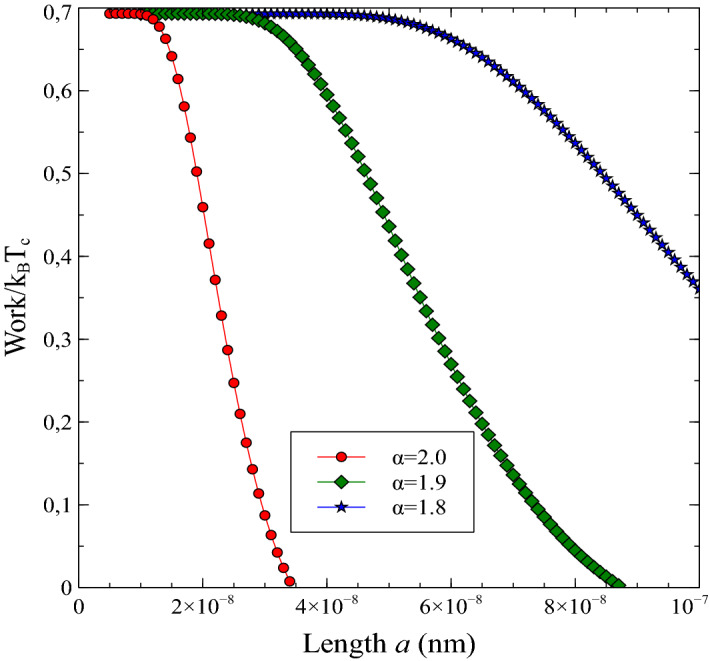
Work *W* of the space-fractional quantum heat engine versus length *a* (in nano-meters) for the various fractional exponents. Here, we have set the constants as $m=9.11\times 10^{-31}$ kg, $k_{B}=1.380649\times 10^{-23}$ J/K, $h=6.62607015\times 10^{-34}$ Js, $T_{h}=2$ K and $T_{c}=1$ K.

**Figure 4:**
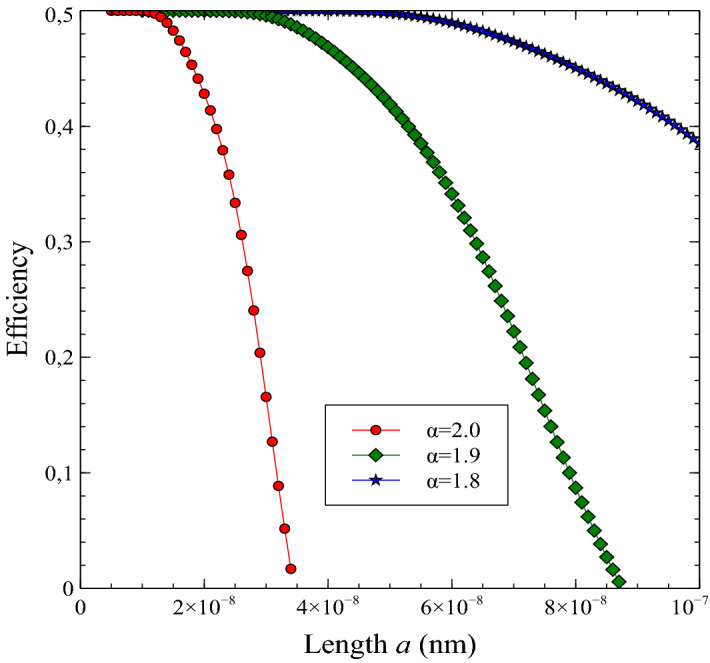
Efficiency $\eta$ of the space-fractional quantum heat engine versus length *a* (in nano-meters) for the various fractional exponents. Here, we have set the constants as $m=9.11\times 10^{-31}$ kg, $k_{B}=1.380649\times 10^{-23}$ J/K, $h=6.62607015\times 10^{-34}$ Js, $T_{h}=2$ K and $T_{c}=1$ K.

As expected obtained numerical results for $\alpha =2.0$ in Fig. 2 are compatible with the results in Ref. ^23^ which confirms our numerical method in the presented study. However, we will see that these solutions clearly deviate in the case of the fractional well as seen from Figs. 3 and 4.

To discuss the effect of the fractional parameter on the work and efficiency we examine the solutions of Eq. (20) and Eq. (21) for the $\alpha <2.0$.

Firstly, we presented the works curves versus the well width parameter *a* for various fractional exponent $\alpha$ in Fig. 3. One can see from the figure that work *W* is the positive and maximum value of work is compatible with the result of Fig. 2 and the result in Ref. ^23^. As expected the extracted work smoothly decreases and reaches zero when well length *a* is increased for various fractional exponent values. Interestingly, we see that while the maximum value of the extracted work for $\alpha =2$ which denote the non-fractional case rapidly decay to zero, however, in the fractional case, the maximum value of the work shifts to the right and persistently lives over longer distances *a*. Furthermore, the work value slowly decreases for the small values of the fractional exponent. This provides that fractional exponent plays a substantial role in the operating range (width of the well) of the quantum heat engine. This result indicates that the size of the quantum device can be enlarged by the fractional deformations.

Secondly, we presented the efficiency curves versus the well length parameter *a* for various fractional exponent $\alpha$ in Fig. 4. One can see similar behavior in the efficiency curves. In fact, the maximum value of the efficiency for $\alpha =2$ is compatible with the results of Fig. 2 and in Ref. ^23^. As well the work behavior, the efficiency also decreases with increasing *a* values. However, the maximum value of the efficiency shifts to the right and persistently lives over longer distances *a*. This behavior also indicates that fractional exponent changes the operating range of the quantum engine. In Fig. 4, the maximum value of the efficiency corresponds to the Carnot efficiency $(1-T_{c}/T_{h})$ which can be obtained at the low-temperature limit. It can be seen from Fig. 4 that this limit can be obtained for a small *a* value for the non-fractional case, however, this limit is attained for large *a* values for the fractional case depends on the fractional exponent. This result also provides that the Carnot limit can keep when the size of the engine is enlarged by using this approximation, at least, for the presented engine which work extracted depends on quantum features.

In summarize, in the present study, we obtain the work and efficiency of the space-fractional quantum engine with a single-particle for infinite well based on energy degeneracy. We show that the fractional exponent plays an important role on the work and efficiency of changing the operating range of the engine.

## Conclusion

The classical and quantum version of the Szilard engine has been studied in detail theoretically and experimentally^1–24^. These studies prove that it is possible to set quantum Szilard heat engines which can extract positive work in nano-size without violate the second law of thermodynamics. Furthermore, these studies verify the connection between thermodynamic entropy and information entropy. It is known that the Szilard engine converts information into useful work and hence measurement is needed to extract work. However, unlike Szilard quantum engine, a Stirling-like engine containing a single or more particle can produce positive work due to quantum features such as energy degeneracy^23^. This engine is a modified version of the Szilard engine, however, it operates in the only quantum regime.

In this study, we consider the fractional counterparts of this Stirling-like engine^23^. In our model, it is supposed that the box is of the space-fractional properties which can emerge due to spatial deformation, entropic restrictions, or deformed potentials. We compute the work and efficiency for various fractional exponents. It is to be noted that the fractional exponent can not be changed during the process. However, before setting the system, the value of this parameter can be tuned by deforming the potential in the system or by making spatial deformations in the system. Therefore, we conclude that this exponent can be used as the controllable parameter.

We show that the fractional exponent plays a crucial role in the work and efficiency. The energy eigenvalues of the particle change because of fractionality and it affects the thermodynamic cycle of the engine. Therefore, it leads to different and interesting results. Indeed, our numerical results show that the work and the efficiency of the space-fractional quantum heat engine which has a Stirling-like cycle depend on the length of the potential as well in the non-fractional case. Additionally, the operating range (width of the well) of the quantum heat engine can be changed by the fractional exponent. Furthermore, the efficiency of the presented engine approach to the Carnot value at the low-temperature limit for the fractional case. However, we see that the efficiency of the fractional engine remains a constant value for large *a* values and slowly decreases more than the non-fractional ones. This can imply that the size of the engine can be enlarged in the nano-scale by using the presented method. In particular, this point can be remarkable for technological applications.

As a result, in this study, we have not only shown that fractional deformations, in space or time, play an important role on the work and efficiency of the quantum heat engines but also introduced the concept of fractional quantum heat engines to the literature.
